# Combinatorial Delivery of Dual and Triple TLR Agonists via Polymeric Pathogen-like Particles Synergistically Enhances Innate and Adaptive Immune Responses

**DOI:** 10.1038/s41598-017-02804-y

**Published:** 2017-05-31

**Authors:** Ranjna Madan-Lala, Pallab Pradhan, Krishnendu Roy

**Affiliations:** The Wallace H. Coulter Department of Biomedical Engineering Georgia Institute of Technology and Emory University, Atlanta, GA 30332 USA

## Abstract

Despite decades of research very few vaccine-adjuvants have received FDA approval. Two fundamental challenges plague clinical translation of vaccine-adjuvants: reducing acute toxicities that result from systemic diffusion of many soluble adjuvants, and delivering multiple adjuvants at the same time to mimic the synergistic immune-stimulation of pathogens, while being safe. In order to address these barriers, we evaluated combinations of four clinically relevant immune-agonists, specifically Toll-like receptor (TLR) ligands, using biodegradable, polymer microparticles. We tested them alone and in combinations of 2 or 3, for a total of 10 unique conditions. We evaluated primary bone-marrow-derived Dendritic Cell phenotypes and functionality, and identified several synergistic combinations. We picked a dual and a triple adjuvant combination, TLR4/TLR9 and TLR4/TLR7/TLR9, for further evaluation and found that both combinations promoted antigen cross-presentation *in vitro*. Studies in mice using the model antigen Ovalbumin, showed that both combinations enhanced lymph node germinal center and T follicular helper cell responses. The triple adjuvant combination showed increased antigen-specific antibody titer with an overall balanced Th1/Th2 response, while the dual combination promoted Th1-polarized IgG responses. Our results show how polymeric particulate-carriers can be adopted to safely deliver combinatorial adjuvants and selectively synergize specific types of immune responses for vaccine applications.

## Introduction

Vaccine development remains largely an empirical process. Currently there are very few adjuvants licensed for use with human vaccines, and these are not able to induce the diverse immune responses required to protect against chronic and emerging diseases^1^. It is critical to develop new adjuvants that are safe, and provide varied immunomodulatory characteristics to enable fine-tuning of disease-specific immune responses. For example, there has been considerable effort towards developing vaccines that induce cellular immunity, which is needed to protect against intracellular pathogens^2–4^. In order to develop customized multicomponent vaccines with specific immune-function, rational design of adjuvants and adjuvant-delivery strategies is critical^5^.

Adjuvants operate by engaging innate immune cells, such as dendritic cells (DCs), and shape the subsequent adaptive immunity^6–8^. Pathogen associated molecular patterns (PAMPs) and danger signals are potent adjuvants; they activate DCs by stimulating cell-surface or intracellular pathogen recognition receptors (PRRs), causing enhanced levels of surface costimulatory markers, e.g. CD86 and CD40, and key secretory cytokines, such as IL12 and IL10, that play a central role in shaping the ensuing adaptive immune responses^9, 10^. Differentially activated DCs help polarize the T helper (Th) cell response, for example to Th1 or Th2, which further guide the nature of cell-mediated or humoral immune responses^9, 11^. Toll like receptors (TLRs) are one the most characterized members of the PRR family^12, 13^. There are ten (human) or twelve (mouse) different TLRs, which are expressed on the cell surface or intracellular compartments of a number of immune cells, including DCs^12, 14–16^. Lipopeptides and peptidoglycans (TLR1, −2, and −6), lipopolysaccharides (TLR4), flagellin (TLR5), dsRNA (TLR3), ssRNA (TLR7 and −8), and CpG DNA (TLR9) are some of the key TLR agonists that have been identified and extensively studied^15, 17–21^.

Studies have shown that a subset of TLR agonists improve the immune response of vaccines in preclinical models and could have great promise in clinical settings^9, 22–25^. For example, the TLR2/TLR1 agonist Pam3CSK4, which is a synthetic triacylated lipopeptide, promotes DC activation, B cell and T cell responses^25^. It has been shown to enhance the immune response to influenza subunit vaccine^26^. The TLR4 agonist, a detoxified derivative of microbial endotoxin lipopolysaccharide, Monophosphoryl lipid A (MPLA) induces potent Th1 or balanced Th1 and Th2 responses^27^. It has shown immense potential and is being evaluated in a number of vaccine formulations in combination with other adjuvants, e.g. AS01, AS02 and AS04^27–30^. Imidazoquinolines (e.g. imiquimod/R837 and resiquimod/R848) are synthetic small molecules that act as ligands for TLR7 and TLR8, and have been extensively evaluated in clinical settings^25, 31, 32^. R837 in topical formulations has been shown to promote antigen specific T cell and antibody responses of a number of vaccines^25, 33–36^. Oligodeoxynucleotides with specific CpG motifs act as TLR9 agonists, many of which are being tested in several preclinical and clinical studies with infectious diseases and cancer^37–40^. CpG has been shown to promote strong Th1 and humoral responses in various models e.g. against malaria, influenza or melanoma specific antigens^25, 41, 42^.

Pathogens harbor multiple PAMPs, and in natural infections immune responses are amplified by several PAMPs presented simultaneously on particulate pathogens (e.g. viruses or bacteria). In order to recapitulate this for generating optimal immune responses for vaccines, combining multiple PAMPs for adjuvant design has been an important strategy in recent vaccine research^9, 24^. Simultaneous triggering of multiple PRRs, such as TLRs, with specific agonists has been shown to activate them synergistically, and lead to enhanced cytokine secretion, T cell and antibody responses^24, 43–46^. Such combinatorial agonists that promote defined immune responses can be leveraged for better vaccine design^24, 43, 47, 48^.

However, a major challenge in translating these promising results into clinical product has been the use of soluble agonists. It is now widely accepted that there are several deficiencies associated with these^49–52^. For example, small molecule adjuvants and hydrophilic adjuvants are easily diffusible and can quickly move from the site of administration into systemic circulation, causing cytokine surge and severe adverse immune-toxicity effects^53, 54^. This becomes even more challenging when multiple adjuvants are used *in vivo*. Early and differential diffusion of soluble adjuvants from their site of administration also limit their effective dose and the adjuvant/antigen ratio, thus preventing optimal interaction of specific adjuvant combinations with immune cells at the site of injection or at the draining lymph nodes (dLNs), key anatomical niches for generating effective vaccine responses. For example, combinations of specified dose and ratios of hydrophilic molecules (e.g. CpG) with hydrophobic adjuvants (e.g. MPLA), that have been optimized using *in vitro* studies in confined tissue culture wells where diffusion away from the application “site” is not an issue, is essentially meaningless *in vivo* due to their different retention kinetics resulting in altered adjuvant ratios and presentation to immune cells.

These issues of systemic immunotoxicity and differential retention can be resolved by the use of biomaterial-based controlled delivery systems, especially particulate carriers. Key advantages of using particle-based delivery include ability to delivery multiple adjuvants to the same innate-immune cell population by localizing defined ratios and mass of adjuvants at the injection site, control adjuvant density and adjuvant-cell ratios, ability to easily deliver multiple adjuvants in a plug-and-play manner, and ability to mimic particulate pathogens in terms of size, shape, adjuvant content, etc. Few studies have already shown that biomaterial-based carriers are superior to soluble adjuvants, and might allow effective simultaneous delivery of multiple adjuvants^55–58^.

In this study we chose to explore how dual and triple adjuvants in previously unexplored combinations and formulations effect immune-stimulation and whether synergistic immune-activation can be achieved. Specifically, we investigated dual and triple combinations of polymeric pathogen-like particle (PLP) formulations of four well-established and clinically relevant TLR agonists, i.e. Pam3CSK4, MPLA, R837 and CpG, for their effects on DC phenotype and functions, and antigen specific adaptive immune responses *in vitro* and *in vivo*. We chose poly(lactic-co-glycolic acid) (PLGA) as our PLP carrier because of its widespread use among the functional materials community, and because it has been extensively characterized and has long been established as an effective delivery vehicle for various biomolecular agents^59–61^. It is already used in FDA approved products for use in humans and would be considered a favorable material for regulatory approval into clinical settings^59^. Our findings suggest that synergistic immune-stimulation can be achieved using combinatorial controlled delivery of these adjuvants and that specific adjuvant combinations can be used to generate unique immune polarizations *in vivo*. These results make significant contributions to the current knowledge on the effects of combinatorial adjuvants on immune response, and highlight the importance of material-based formulations to deliver specific adjuvant combinations and elicit desired immune responses.

## Results

### Synthesis and characterization of PLPs

To investigate the effects of specific combinations of well-established TLR agonists on immune response, we fabricated PLPs with PLGA by water-oil-water double emulsion method, as reported previously^62–64^. The particles were loaded with Pam3CSK4, MPLA and R837, agonists for TLR2, −4 and −7 respectively, by encapsulation, or by surface modifying them with polyethyleneimine (PEI) to generate cationic particles (zeta potential 37.57 ± 4.8 mV), followed by electrostatic adsorption of CpG, TLR9 agonist, or model antigen Ovalbumin, as described previously and in methods^55, 63, 64^. To facilitate flexibility in using different combinations of two or three adjuvants, in this study we used PLPs loaded with single adjuvants. Table 1 shows characterization of these PLPs for their size, zeta potential, loading method and efficiency.

**Table 1:** Size, Zeta potential, and representative encapsulation/loading efficiency of PLGA-microparticles (PLP).

Formulation	Size (μm)	Zeta Potential (mV)	Loading Method	Loading Efficiency
PLGA-Pam3CSK4	1.5 ± 0.14	−7.96 ± 0.68	Encapsulation	6.4 μg/mg (69.9 ± 0.9%)
PLGA-MPLA	1.69 ± 0.29	−7.59 ± 0.15	Encapsulation	12.5 μg/mg (100%)
PLGA-R837	1.79 ± 0.29	−6.82 ± 0.13	Encapsulation	7.7 μg/mg (30.5 ± 1.3%)
PLGA-PEI-CpG	1.72 ± 0.37 (before PEI modification)	−20.56 ± 4.62	Surface Loading	10.4 μg/mg (86.6 ± 12%)
PLGA-PEI-Ova	1.72 ± 0.37 (before PEI modification)	18.43 ± 2.09	Surface Loading	25.8 μg/mg (51.7 ± 3.0%)

The data shown are average of at least 2 (Pam3CSK4), 3 (Ovalbumin) or 5 (MPLA, R837, and CpG) batches.

### Specific combinations of PLPs act synergistically to modulate DC activation

In the following studies comparison with soluble adjuvants, either alone or in combination, were not made. As discussed in the Introduction section, soluble combinations have little clinical or *in vivo* relevance due to acute systemic toxicities of many soluble adjuvants (e.g. CpG) that prevent clinical use. Furthermore, soluble combinations optimized *in vitro* are also not relevant for *in vivo* applications due to differential retention kinetics at the site of injection. Therefore, combination adjuvants only in the context of PLPs are compared in this study.

**Figure 1:**
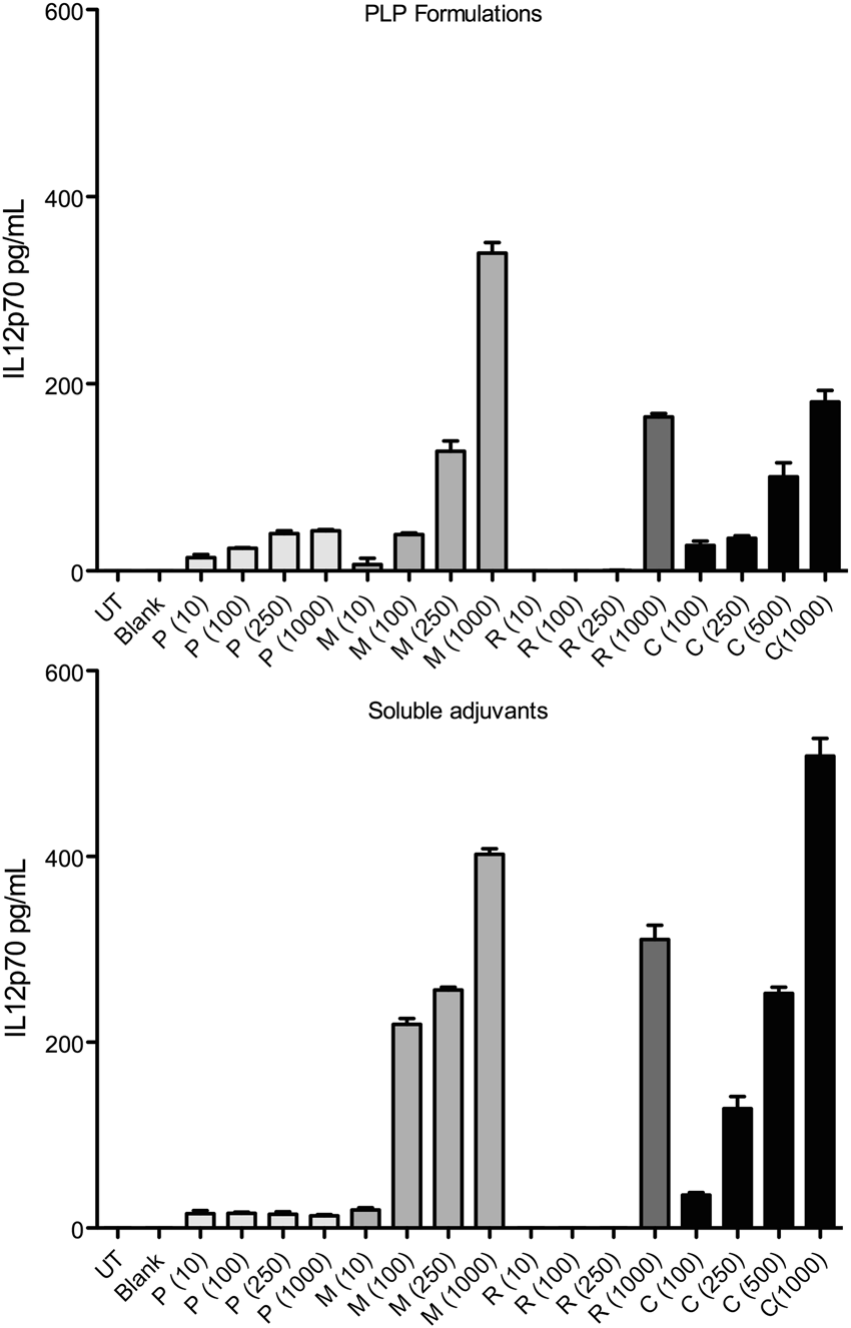
Adjuvant dose response study. BMDCs derived from C57BL/6 J mice were exposed to medium alone (UT) or treated with blank PLP (Blank) or increasing doses of PLP formulations or soluble Pam3CSK4 (P), MPLA (M), R837 (R) (10 ng mL^−^, 100 ng mL^−^, 250 ng mL^−^, or 1000 ng mL^−^) or CpG (C) (100 ng mL^−^, 250 ng mL^−^, 500 ng mL^−^, or 1000 ng mL^−^). At 24 h post exposure, cell-free supernatants were harvested and assayed for IL12p70 by ELISA. Data are represented as mean ± SEM of 4 replicates.

To evaluate the effects of different PLP combinations on DC activation, we first defined sub-saturating dose for each PLP, in order to avoid saturating the individual TLR signaling pathways with high single adjuvant doses, which might prevent detection of any potential combinatorial effects of these adjuvants. We studied dose response for each adjuvant by stimulating BMDCs with increasing doses of soluble or PLP formulations of Pam3CSK4 (P), MPLA (M), R837 (R), and CpG (C). Supernatants isolated at 24 h post stimulation were assayed for IL12p70 by ELISA (Fig. 1). Based on these findings, we selected sub-saturating PLP doses (100 ng mL^−^, 10 ng mL^−^, 1 μg mL^−^ and, 100 ng mL^−^ for P, M, R and C respectively) for subsequent experiments.

**Figure 2:**
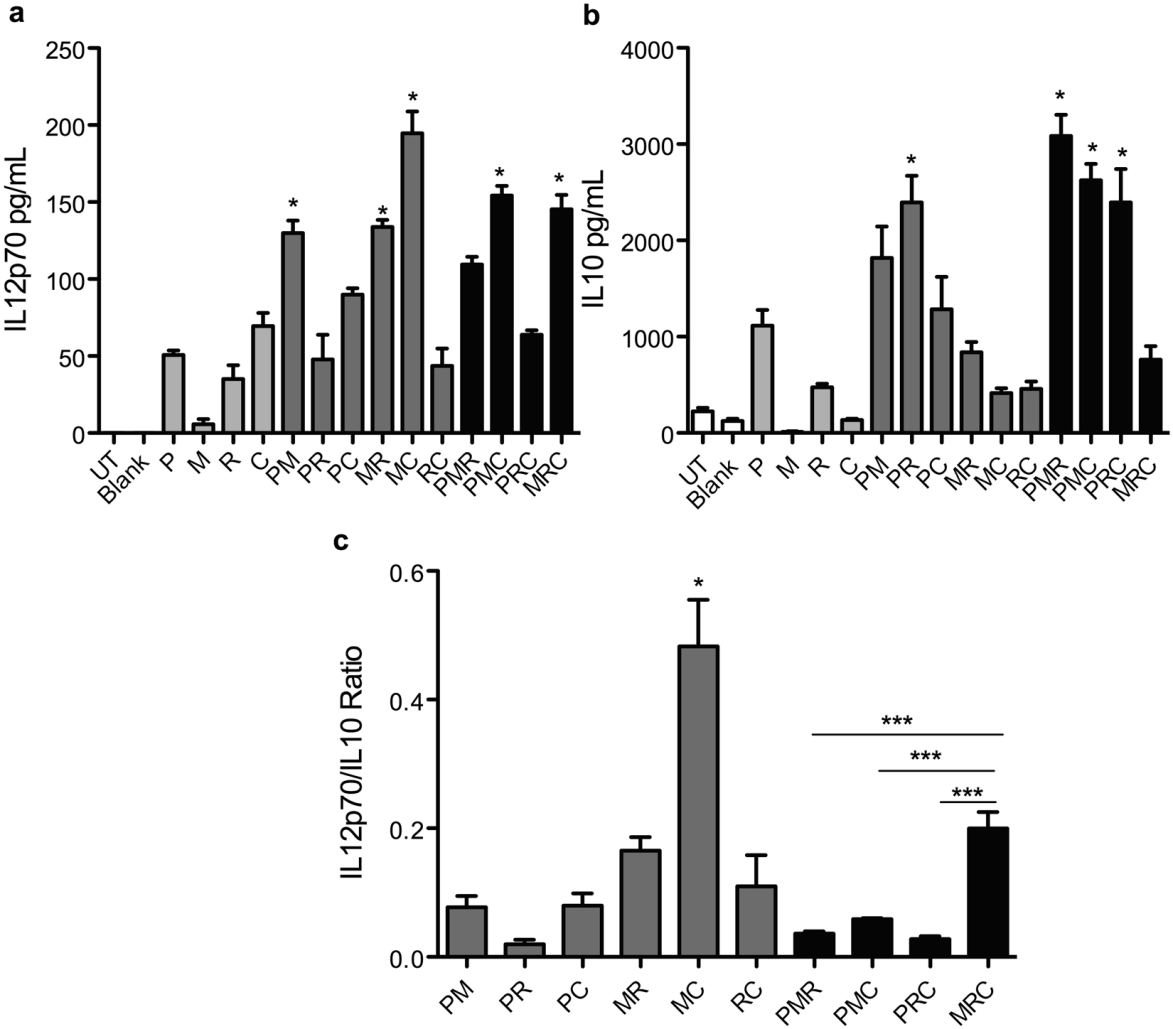
Synergistic stimulation of IL12p70 and IL10 production by different PLP combinations. BMDCs were exposed to medium alone (UT) or treated with blank PLP (Blank), 100 ng mL^−^of PLP-Pam3CSK4 (P), 10 ng mL^−^ of PLP-MPLA (M), 1 μg mL^−^ of PLP-R837 (R), or 100 ng mL^−^ of PLP-CpG (C), individually or in combinations of 2 or 3 for 24 h. Cell-free supernatants were harvested and assayed for (**a**) IL12p70 or (**b**) IL10 by ELISA. Ratio of IL12p70 to IL10 for each combination is shown in (**c**). Data from one representative experiment out of three are shown as mean ± SEM of 3 replicates. Statistical analysis was performed by 1 way Anova followed by Tukey’s multiple comparison tests. (**a** and **b**) *Represents significant and synergistic differences from individual components. (**c**) *Represents significant difference (p < 0.05) from all groups. ***(p < 0.0001).

To examine the effects of simultaneous stimulation of TLR2, −4, −7 or −9 by using dual or triple combinations of the respective PLPs on DC activation, we exposed BMDCs to media alone or individual PLPs (P, M, R or C) or their double (P+M; P+R; P+C; M+R; M+C; R+C) or triple (P+M+R; P+M+C; P+R+C; M+R+C) combinations. Cell-free supernatants were collected at 24 h post exposure, and assayed for IL12p70 by ELISA (Fig. 2a). As shown in Fig. 2a, P+M, M+R, M+C, P+M+C, and M+R+C PLP combinations elicited significantly higher IL12p70 as compared to the individual PLPs, and the IL12p70 synergy ratio (calculated as described in methods) for these combinations was 2.1, 2.9, 2.4, 1.2 and 1.2 respectively. We also measured IL10 in the culture supernatants by ELISA. As shown in Fig. 2b, PLP combinations P+R, P+M+R, P+M+C and P+R+C induced significantly higher IL10 than the individual adjuvants, and the IL10 synergy ratios for these combinations was 1.5, 1.9, 2.1 and 1.4 respectively. IL10 synergy ratio for combinations that synergistically enhanced IL12p70, i.e. P+M, M+R, M+C, P+M+C, and M+R+C, was 1.6, 1.7, 2.8, 2.1 and 1.2 respectively. We decided to select two combinations for further studies into their potential to promote specific Th polarization phenotypes. Thus, we defined the ratio of IL12p70 to IL10 elicited by these combinations, which is a predictor of Th1/Th2 polarizing responses (Fig. 2c). As seen in Fig. 2c, M+C combination had the highest IL12p70 to IL10 ratio (0.48). Of the triple combinations, M+R+C showed the highest IL12p70 to IL10 ratio (0.19), and we used this combination for further in depth investigation.

**Figure 3:**
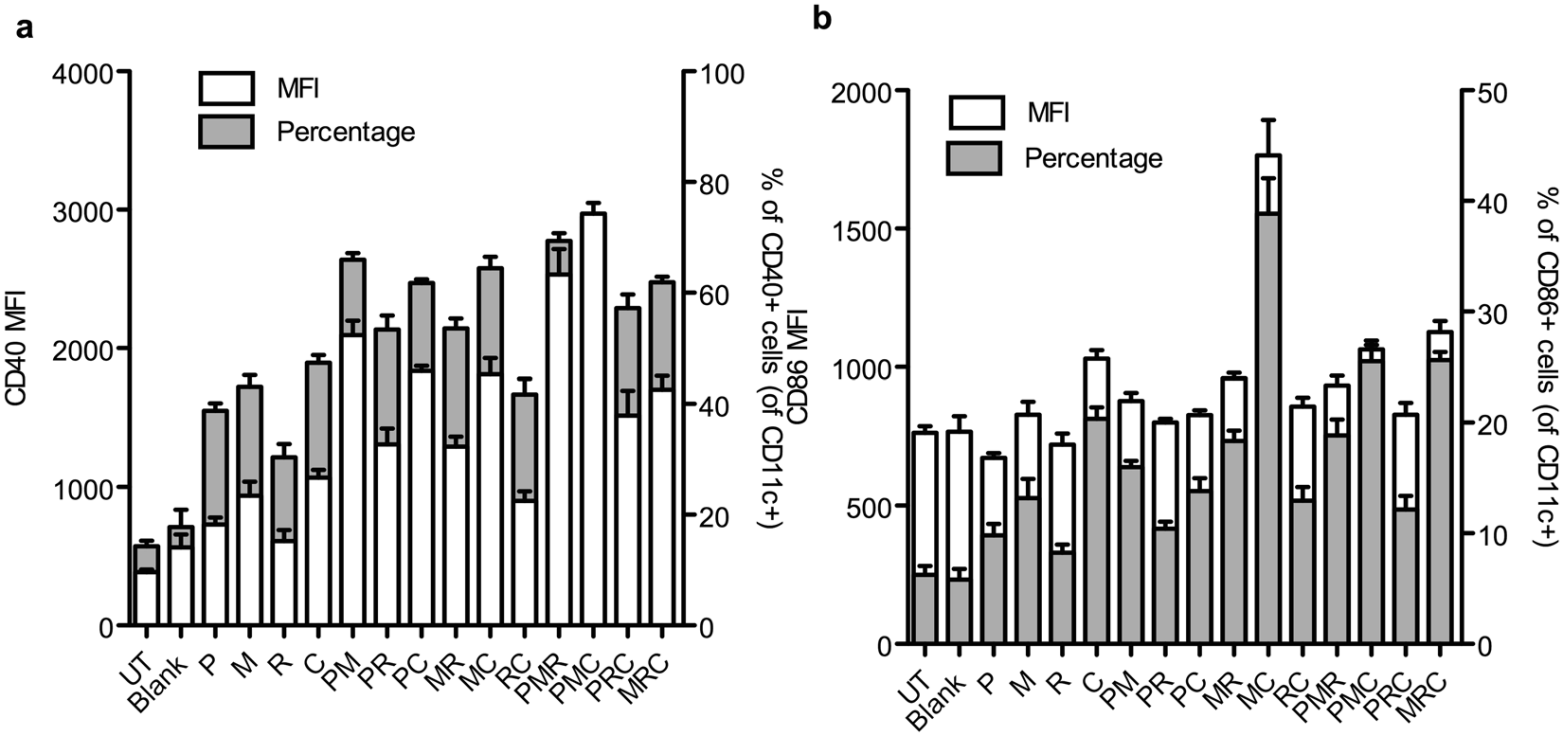
Stimulation of cell surface BMDC activation markers by different PLP combinations. BMDCs were exposed to medium alone (UT) or treated with blank PLP (Blank), 100 ng mL^−^ of PLP-Pam3CSK4 (P), 10 ng mL^−^ of PLP-MPLA (M), 1 μg mL^−^ of PLP-R837 (R), or 100 ng mL^−^ of PLP-CpG (C), individually or in combinations of 2 or 3 for 24 h. Cells were labeled with anti CD11c, anti CD40, and anti CD86. Percentage and MFI of CD40+ cells (**a**) and CD86+ cells (**b**) on CD11c+ cells is shown. Data from one representative experiment out of three are shown as mean ± SEM of 3 replicates. Statistical analysis was performed by 1 way Anova followed by Tukey’s multiple comparison tests.

Further, we also analyzed these BMDCs for their surface activation markers, CD40 and CD86 by flow cytometry. Median fluorescence intensity (MFI) and percentage of co-stimulatory markers CD40 and CD86 on CD11c+ gated cells were measured (Fig. 3a and b). All PLP combinations elicited statistically significantly difference (p < 0.05) in MFI and percentage of CD40 and CD86 as compared to their individual components, except P+M and P+M+C, which did not induce significantly different CD86 MFI as compared to M or C individually; and P+R for which percentage of CD86 cells was not significantly different than P alone.

### M+C and M+R+C combinations enhance antigen cross presentation ***in vitro***

Robust antigen presentation by APCs is critical for generation of adaptive immune response. The results reported above indicate that PLP combinations of M+C and M+R+C synergistically induce IL12p70, and modulate the Th1/Th2 shaping cytokine levels (Fig. 2). To examine the ability of these adjuvant combinations on priming and polarization of T cells, we co-cultured BMDCs stimulated with these PLPs with TCR-Tg CD4 T isolated from OT-II mice for 72 h. Cell free culture supernatants were assayed for IFNγ and IL4. Surprisingly, we did not observe significant Th1 or Th2 bias since DCs exposed to M+C or M+R+C combinations did not specifically promote higher IFNγ or IL4 production by T cells or higher T cell proliferation as compared to single PLPs (Supplementary Fig. S1).

**Figure 4:**
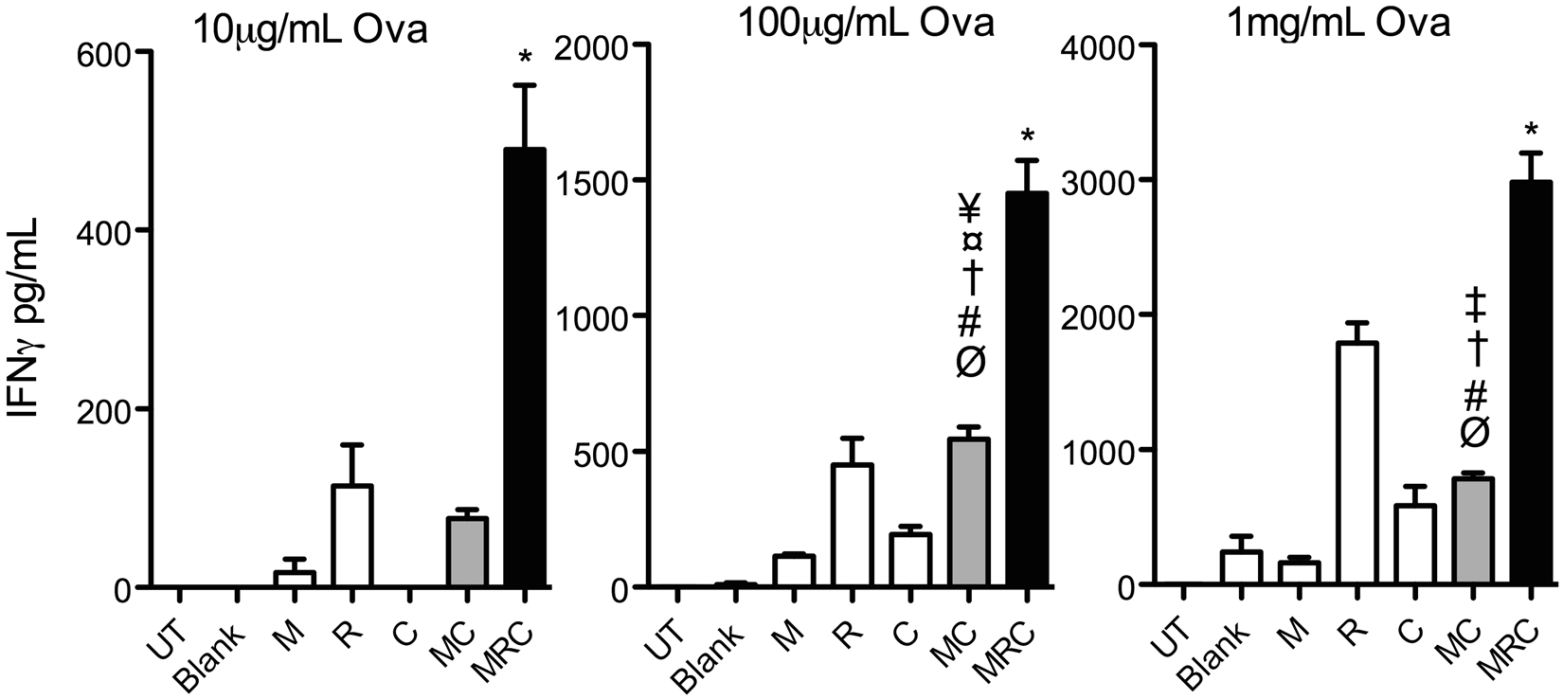
MC and MRC combinations augment antigen cross presentation. BMDCs in medium alone (UT) or exposed to blank PLP (Blank), 10 ng mL^−^ of PLP-MPLA (M), 1 μg mL^−^ of PLP-R837 (R), or 100 ng mL^−^ of PLP-CpG (C), individually or in combinations of M+C or M+R+C in the presence of soluble Ovalbumin were co-cultured with OTI CD8 T cells at 1:4 ratio for 72 h. Cell free supernatants were harvested and assayed for IFNγ by ELISA. Data are represented as mean ± SEM of 4 or 5 replicates. Statistical analysis was performed by 1 way Anova followed by Tukey’s multiple comparison tests. The following symbols indicate significant differences (p < 0.05) with different groups: *(All); ^#^(UT); ^¤^(Blank); ^†^(M); ^‡^(R); ^¥^(C); ^$^(MC); ^Ø^(MRC).

Next, we tested the ability of these BMDCs for their capacity for antigen cross presentation by co-culturing DCs stimulated with PLPs in the presence of increasing soluble antigen (Ovalbumin) doses, with TCR-Tg CD8 T isolated from OT-I mice for 72 h. While we did not see significant effects of the combinatorial PLPs on CD8 T cell proliferation as measured by CFSE dilution assay (Supplementary Fig. S2), DCs matured with M+R+C PLP combinations were more efficient at antigen cross presentation amongst all groups tested at all antigen doses, M+C combination promoted better antigen cross presentation than M and C at specific antigen concentrations (Fig. 4).

### Vaccinations with M+C and M+R+C PLPs promote enhanced humoral response

**Figure 5:**
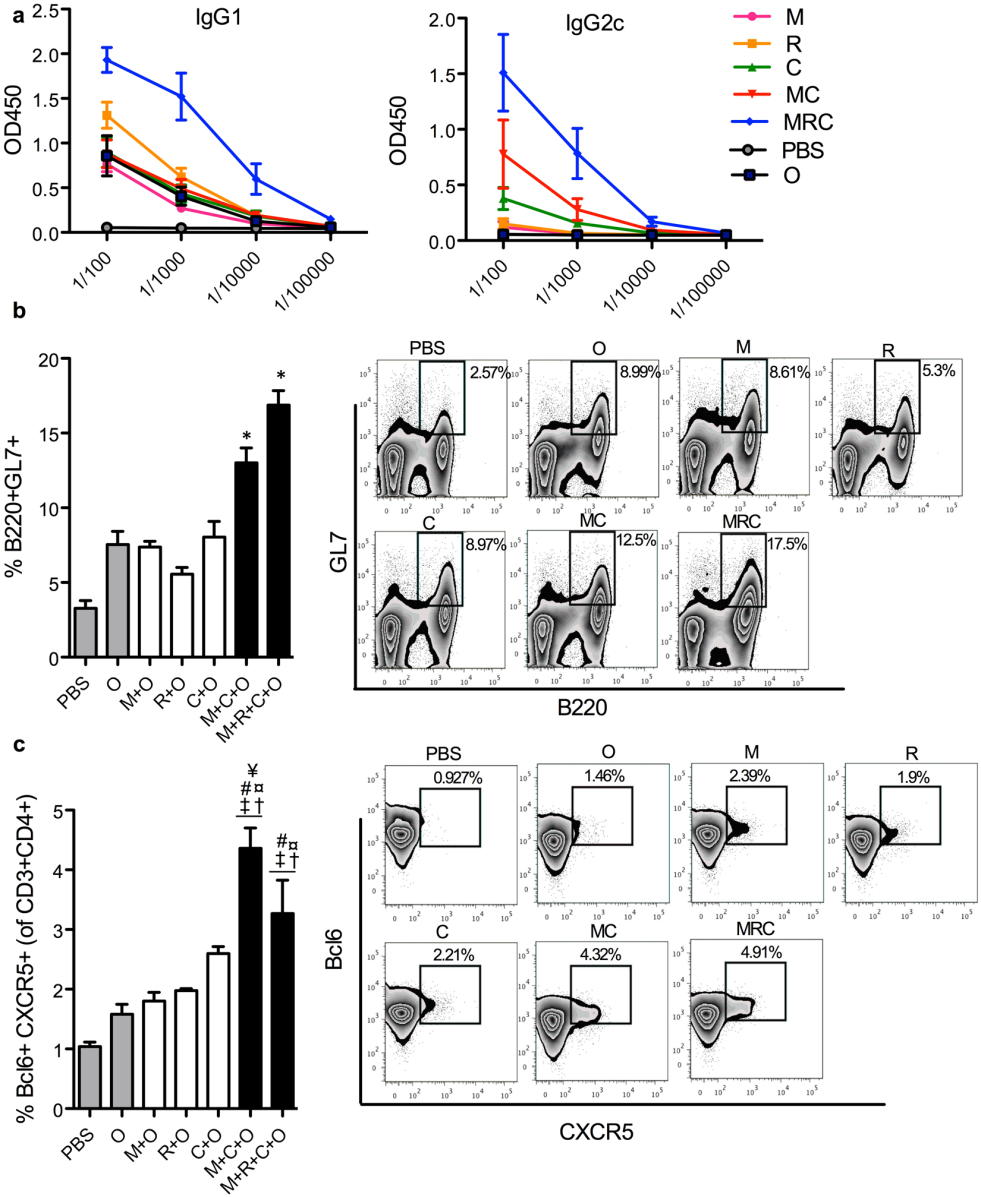
MC and MRC combinations promote robust humoral responses. Mice were immunized s.c. with PBS, PLP-Ova (O), with or without PLP-M, R, C, M+C, or M+R+C. At day 35, draining lymph nodes and blood sera were analyzed. (**a**) Ova specific IgG1 and IgG2c response in sera is depicted. Graphs and a representative figure from each group showing frequencies of germinal center (**b**) and Tfh cells (**c**) in draining lymph nodes are shown. Data are represented as mean ± SEM of at least 5 replicates. Statistical analysis was performed by 1 way Anova followed by Tukey’s multiple comparison tests. (**b** and **c**): The following symbols indicate significant differences (p < 0.05) with different groups: *(All); ^#^(UT); ^¤^(Blank); ^†^(M); ^‡^(R); ^¥^(C); ^$^(MC); ^Ø^(MRC).

We next tested how M+C and M+R+C PLP combinations affect antigen specific adaptive immune responses *in vivo*. C57BL/6 J mice were immunized with Ovalbumin-PLP with or without the PLPs M, R, C, M+C or M+R+C at day 0, weeks 2 and 4. Blood sera were collected at week 5 post 1^st^ vaccination and assayed for IgG1 and IgG2c by Ova specific ELISA, as described in methods. As shown, M+R+C PLP combination increased the production of IgG1 and IgG2c antibodies, while mice vaccinated with M+C combination had high levels of IgG2c, but not IgG1 (Fig. 5a), as compared to the respective individual PLPs, suggesting a Th1 polarized response. Next, we investigated the effects of combinatorial adjuvants on germinal center (GC) formation in the lymph node (site of activated B cell proliferation, somatic hypermutation and immunoglobulin class switching) and T follicular helper cell (Tfh) response, which provide crucial help for humoral response. Flow cytometry analyses of cells from draining lymph nodes harvested at day 35 post immunization revealed that both PLP-combination vaccinated mice had significantly higher B220+GL7+ activated germinal center B cells (Fig. 5b). We also saw an enhanced Tfh response in the lymph nodes as measured by an increase in CXCR5+Bcl6+ cells (gated on CD3+CD4+) in draining lymph nodes (Fig. 5c), suggesting that the enhanced antibody levels elicited by the combinatorial adjuvants may be due to higher germinal center and Tfh response.

## Discussion

Emerging evidence demonstrates the ability of specific combinations of adjuvants to synergistically enhance vaccine induced immune response. However, most research in this field has focused on using soluble adjuvants, which have several disadvantages; for example, their potential to diffuse systemically can result in serious side effects^50, 54^. Furthermore, differences in retention kinetics of small molecules with different hydrophilicity and hydrophobicity severely restrict the adjuvant combinations that can be used effectively with predicted outcomes. Biomaterial carrier based delivery of adjuvants has proven to be an effective strategy for diminishing their toxic effects and improving the overall efficacy^65, 66^. In this study, we carried out an extensive comparison of PLGA microcarrier based dual and triple combinations of TLR2, −4, −7 and −9 agonists for their potential to enhance specific immune responses *in vitro* and *in vivo*. Given the well established and previously described limitations of using soluble adjuvants, which make them clinically irrelevant especially for multi-adjuvant delivery, we deliberately decided not to include any soluble agonists in our study, and focused on comparing the effects of various PLP combinations. We exposed DCs to individual, dual or triple PLP combinations, and tested how they modulate DC responses and the subsequent adaptive immunity. Our findings reveal new insights into the effects of specific combinations of four highly clinically relevant TLR adjuvants on immune response, and highlight the potential of two specific PLP combinations, M+C and M+R+C, in inducing enhanced antigen cross presentation and humoral immune responses.

IL12p70 and IL10 are key inflammatory cytokines that are critical in polarizing Th response to Th1 or Th2; and Th1/Th2 balance determines the overall nature of the adaptive immune response. IL12p70 promotes Th1 biased immune responses^67^, while IL10 boosts Th2 oriented immune responses, and dampens the Th1 response. Thus, we decided to primarily focus on assaying IL12p70 and IL10 secretion by DCs, and evaluated the quantitative and qualitative differences in induction of these cytokines by various PLP combinations. Our results showed that PLP combinations of P+M, M+R, M+C, P+M+C, and M+R+C led to synergistic enhancement IL12p70 secretion over their individual components. P+R, P+M+R, P+M+C and P+R+C significantly and synergistically increased IL10 induction. We found that M+C combination significantly altered the IL12p70/IL10 ratio, as well as synergistically enhanced the IL12p70 levels. Most of the research on combinatorial TLR adjuvants has focused on using dual combinations. For example, combination of MPLA and CpG was shown to induce significantly higher T cell responses against a Leishmania vaccine candidate as compared to single adjuvants^45^. Another study has shown that polymeric particles carrying ligands for TLR4 and TLR7 synergistically enhance antigen specific production as compared to either single ligand carrying particles^55^. Very little is known about the effects of triple TLR agonist combinations on modulating the immune response; however, simultaneous triggering of more than two TLRs has great potential in enhancing selective immune responses. A study has shown that combining agonists for TLR2, −3, and −9 greatly improves HIV envelope peptide vaccine response in mice^68^, underscoring the importance of combining more than two TLR agonists to synergistically boost the immune response. Hence, in addition to a dual combination, we also studied a triple combination to ascertain its potential in modulating the immune responses. Amongst the triple combinations, M+R+C significantly altered the IL12p70/IL10 ratio, in addition to synergistically enhancing the IL12p70 secretion. Thus, we chose two combinations, i.e. M+C and M+R+C, for further detailed studies on their effects on adaptive immune responses.

Surprisingly, despite a significantly altered BMDC cytokine response as compared to the respective individual adjuvants, M+C and M+R+C PLP combinations did not exhibit any notable difference in promoting CD4 T cell antigen presentation potential by DCs. When we tested the ability of BMDCs for their capacity for antigen cross presentation by co-culturing DCs stimulated with these adjuvants in the presence of different antigen (Ova) doses, with TCR-Tg CD8 T isolated from OT-I mice, we found that both M+C and M+R+C PLP combinations showed enhanced IFNγ secretion, suggesting that these PLP combinations improve cross presentation of soluble antigens. Presentation of exogenous antigens in APCs is mainly carried out via class II MHC pathway, and their class I MHC presentation is very inefficient, resulting in overall weak priming of CD8 T cells. Hence, finding adjuvants that improve antigen cross presentation is really significant, especially for developing vaccines needed to elicit robust cellular immune responses.

Using an Ova immunization model, we showed that both M+C and M+R+C combinations induced potent germinal center and Tfh response in the draining lymph nodes, as compared to the respective individual PLPs. In terms of antibody response, while M+R+C showed an overall increase in antigen specific IgG1 and IgG2c titers, suggesting a balanced Th1/Th2 response, M+C PLPs promoted enhanced IgG2c but not IgG1, indicating that this combination promotes Th1 biased response. Surprisingly, we did not observe significant antigen specific CD4 or CD8 T cell responses in this model (data not shown), which could be a limitation of the antigen dose and the immunization schedule tested for this study, and future studies will be directed with different antigen doses and immunization schedules to test the impact of these adjuvant combinations on T cell responses.

In summary, this study provides significant insights into an array of specific immune responses that can be elicited by using defined adjuvant combinations, and expands our current knowledge on the effects of clinically relevant adjuvants. Our results highlight the capacity of MPLA/CpG and MPLA/R837/CpG adjuvant combinations for enhanced antigen cross presentation and humoral immune responses, and underscore the potential of using PLP based delivery systems to synergistically augment specific immune responses. Overall, these findings can be leveraged for devising specific strategies for vaccine design and immunotherapy applications.

## Methods

### Preparation and characterization of PLGA microparticle formulations

PLPs used in this study were prepared with PLGA using a water-oil-water double emulsion, solvent evaporation method as reported earlier^55, 62–64^. MPLA (detoxified lipid A, Avanti Lipids) was dissolved in chloroform at 5 mg mL^−^, TLR7 ligand R837 (Invivogen) was dissolved at 10 mg mL^−^ in DMSO with heating; Pam3CSK4 (Invivogen) was dissolved in water at 6.6 mg mL^−^. For encapsulating these agonists, 200 mg of PLGA (RG502H, Sigma Aldrich, MO) was dissolved in 7 ml dichloromethane (DCM) (Sigma Aldrich, MO). 250 μl MPLA followed by 150 μl water; 250 μl R837 mixed with 150 ul water; 300 μl Pam3CSK4 or 300 μl water was added to PLGA dissolved in DCM, and homogenized at a speed of 10,000 rpm for 2 min. The emulsion was added to 50 ml of 1% PVA (Sigma Aldrich, MO) and homogenized again for 2 min at 10,000 rpm and stirred for 3.5–4 h for complete evaporation of DCM. The PLPs were collected by centrifugation and washed 3 times with deionized water, followed by lyophilization, and stored at −20 °C until further use. As described previously^55^ R837 encapsulation was estimated by measuring absorbance at 325 nm using a standard curve of R837 in DMSO. Based on hydrophobicity of the molecule and previous publications^55^, MPLA encapsulation was taken to be 100%. Pam3CSK4 encapsulation was estimated by Fluradehyde OPA (Thermofisher Scientific) assay using a Pam3CSK4 standard curve, as per the manufacturer’s protocol. Surface functionalization of the synthesized PLGA microparticles was done by covalent conjugation of branch PEI (MW 70,000, Polyscience, PA) with the acid group on the surface of PLGA microparticles using EDC/NHS chemistry as also reported earlier by our group^62–64^. CpG ODN 1826 (Invivogen, San Diego, CA) or Ovalbumin (EndoGrade Ovalbumin BioVedor, NC) were surface loaded on PLGA-PEI particles by electrostatic interaction. Briefly, PLGA-PEI particles at 5 mg mL^−^ (for CpG) or 5 mg (0.5 mL)^−^ (Ova) and CpG ODN at 60 μg mL^−^ or Ovalbumin at 250 μg (4.5 mL)^−^ were resuspended in PBS in two separate DNase RNase free tubes. The PLGA-PEI solution was added drop-wise to the CpG or Ova solution while vortexing and the mixture was incubated on an end to over shaker overnight at 4 °C. The particles were collected by centrifugation and the supernatant was analyzed for CpG at 260 nm wavelength using a Take 3 micro-volume plate in a BioTek reader (BioTek, VT) and Ova by micro BCA assay (Thermo Scientific, IL). The amount of nucleic acid or protein present in the supernatant was subtracted from the initially added amount to quantify the total amount of surface loaded nucleic acid onto PLGA-PEI microparticles. Particles were characterized for their size and zeta potential using a Zetasizer Nano ZS instrument (Malvern, MA).

### Mice

All animal experiments were approved by the Institutional Animal Care and Use Committee at the Georgia Institute of Technology and all methods were performed in accordance with the relevant guidelines and regulations. Mice were housed under pathogen-free conditions in the physiological research laboratory (PRL) at the Institute of Bioscience Bioengineering (IBB), Georgia Institute of Technology, and provided with sterile water and food ad libitum. C57BL/6 J, OTI Tg mice (specific for Ova_257–264_) were purchased from the Jackson Laboratory.

### BMDCs stimulation and activation assays

Bone marrow cells from C57BL/6 J mice were grown in RPMI 1640 medium (Lonza, Walkersville, MD) with 10% heat-inactivated FBS (HyClone, Logan, UT), 2 mM glutamine, β-ME, 10 mM HEPES, 1 mM sodium pyruvate, 1X nonessential amino acids, and 20 ng mL^−^ murine recombinant GM-CSF (Peprotech, Rocky Hill, NJ) for generating BMDCs. Incubations were carried out at 37 °C with 5% CO2. Fresh medium with GM-CSF was added on days 2, 4 and 6, and cells were used on day 7. 3 × 10^5^ cells per well in 96 well polypropylene plates were stimulated with adjuvants at various concentrations as mentioned in results. Cell free supernatants were assayed for IL12p70 and IL10 by ELISA using kits from Ebiosciences as per the manufacturer’s instructions. To estimate synergy in cytokine induction, synergy ratio was calculated = Amount of cytokine induced by the micro-PLP combination/Sum of total cytokine amounts induced the individual micro-PLPs of that combination. Staining for cell-surface markers (CD11c, CD40 and CD86) was done by resuspending cells in 200 μl FACS buffer (PBS with 2% FBS) with anti-mouse CD16/CD32 Fc block at 4 °C for 10 min, followed by incubation at 4 °C for 30 min with murine anti-CD11c APC, anti-CD40 PE, anti-CD86 FITC antibodies (Ebiosciences). Cells were washed with FACS buffer, stained with 7-AAD viability stain (BD Biosciences) and data were acquired using an BD Accuri or BD LSRII (BD Biosciences). Data were analyzed with FlowJo software (Tree Star, San Carlos, CA).

### Antigen presentation assays

CD8 T cells were purified from single-cell suspensions of spleen from OTI Transgenic mice using the CD8 T cell isolation kit and AutoMACS column as per the manufacturer’s instructions (Miltenyi Biotec). 3 × 10^5^ BMDCs per well in 96 well polypropylene plates were incubated with 10 μg mL^−^, 100 μg mL^−^ or 1 mg mL^−^ soluble Ovalbumin in the presence or absence of various adjuvants at concentrations specified in results for 24 h. DCs were washed with PBS and co-cultured with Ag specific CD8 T cells at a ratio of 1:4 for 72 h. Supernatants collected from these cells were analyzed for IFNγ and IL4 by ELISA using ready-set-go kits (Ebioscience) according to the manufacturers’ instructions.

### Mouse Immunization and tissue harvests

6–8 week old C57BL/6 J mice were immunized with various PLP formulations (10 μg Ova, 37 μg MPLA, 50 μg R837 or 20 μg CpG ODN per injection respectively), subcutaneously (inguinal) in 200 μl volume three times, at 2 weeks interval. PBS or antigen alone injected mice were used as negative controls. Blood serum, spleen and lymph nodes were harvested at 5 weeks post 1^st^ immunization. Serum antibody ELISAs were carried out as described elsewhere^69^ with some modifications: 96 well Nunc maxisorp plates were coated with 100 ng Ova (100 μl)^−^ in carbonate buffer at 4 °C overnight. Plates were washed with washing buffer (PBS + 0.05% Tween20), and blocked with 4% BSA in PBS + 0.05% Tween20 for 1 h at 37 °C. Blocking buffer was removed and blood sera diluted 10 fold serially (100x, 1000x, 10000x, 100000x) in PBS + 0.05% Tween20 were added to the plates, and incubated at 37 °C for 2 h. Plates were washed 5 times with washing buffer and incubated with 5000 fold dilution of IgG1-HRP (Southern Biotech, AL) or IgG2c-HRP (Southern Biotech, AL) at 37 °C for 1 h. Plates were washed and developed using TMB (tetramethylbenzidine) substrate (Southern Biotech, AL) and were stopped using 2 N sulfuric acid. Absorbance was read at 450 nm using a BioTekplate reader. Anti-mouse antibodies for Flow cytometry: GL7 FITC (Biolegend), B220 PerCPCy5.5 (ebiosciences), CD3 PECy7 (Biolegend), CD4 APC (ebiosciences), Bcl6 PE (Biolegend) (intracellular staining), CXCR5 PE/Dazzle (Biolegend) were used in this study. For surface staining single cell suspensions from draining lymph nodes were washed with FACS buffer and stained for 30 min at 4 °C, followed by washing with FACS buffer. For intracellular staining, established protocol from BD Biosciences was used. Briefly, surface stained cells were fixed and permeabilized using Cytofix/Cytoperm (BD Biosciences) and stained for intracellular antibodies in 1x BD perm/wash solution at 4 °C for 30 min. Cells were washed with perm wash followed by FACS buffer. Stained cell samples were fixed with BD Cytofix (BD Biosciences) and acquired on a LSR-II cytometer (BD Biosciences). FACS data were analyzed on Flow JO software.

### Statistics

1 way Anova followed by Tukey’s multiple comparison tests were performed. The following symbols indicate significant differences (p < 0.05) with different groups: *(All); ^#^(Untreated); ^¤^(Blank); ^†^(MPLA); ^‡^(R837); ^¥^(CpG); ^$^(M+C); ^Ø^(M+R+C). Where indicated ***(p < 0.0001).

### Data Availability

All relevant data supporting the results of this study are included in the current article and supplementary information. Raw data and additional supporting data can be made available from the authors upon reasonable request.

## Electronic supplementary material

Supplementary data
