# Supplementary material for: Combinatorial Delivery of Dual and Triple TLR Agonists via Polymeric Pathogen-like Particles Synergistically Enhances Innate and Adaptive Immune Responses
Source: Sci Rep. 2017 May 31;7:2530. doi: 10.1038/s41598-017-02804-y (PMC5451393; doi:10.1038/s41598-017-02804-y)
Supplement: Supplementary file 1 — Supplementary data [file 41598_2017_2804_MOESM1_ESM.pdf]

Combinatorial Delivery of Dual and Triple TLR Agonists via Polymeric Pathogen-like Particles Synergistically Enhances Innate and Adaptive Immune Responses

Ranjna Madan-Lala, Pallab Pradhan, Krishnendu Roy*

The Wallace H. Coulter Department of Biomedical Engineering, Georgia Institute of Technology and Emory University, Atlanta, GA 30332 USA.

***Corresponding author:**
Krishnendu Roy, PhD
Robert A. Milton Chair
Director, Marcus Center for Cell-Therapy Characterization and Manufacturing (MC3M),
Director, Center for ImmunoEngineering at Georgia Tech
The Wallace H. Coulter Department of Biomedical Engineering at Georgia Tech and Emory
The Parker H. Petit Institute for Bioengineering and Biosciences
Georgia Institute of Technology, Atlanta, GA
EBB 3018, 950 Atlantic Dr NW, Atlanta, GA, 30318
Ph: 404-385-6166; krish.roy@gatech.edu

Supplementary Figures

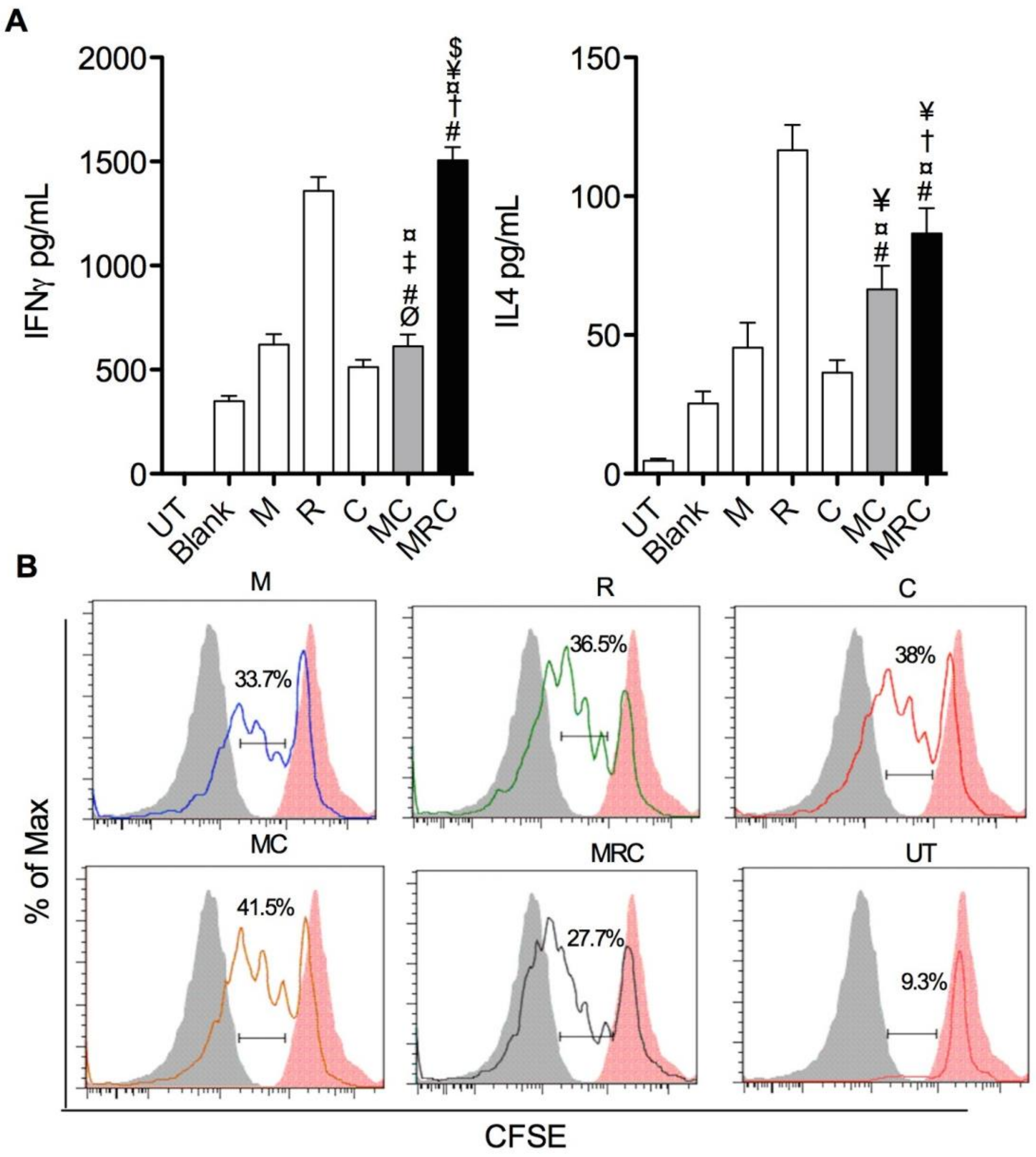

Supplementary Figure S1. CD4 Antigen presentation assay. BMDCs in medium alone (UT) or exposed to blank PLP (Blank), 10 ng mL^{-} of PLP-MPLA (M), 1 µg mL^{-} of PLP-R837 (R), or 100 ng mL^{-} of PLP-CpG (C), individually or in combinations of M+C or M+R+C in the presence of Ovalbumin were cocultured with CFSE labeled OTII CD4 T cells at 1:4 ratio for 72 h. A) Cell free supernatants were harvested and assayed for IFNγ and IL4 by ELISA. B) Cells were stained with antibodies against CD3 and CD4, and analyzed by Flow Cytometry. Unlabeled (gray) and CFSE labeled and untreated (Red) T cells were used as controls. Data are represented as mean ± SEM of 5 (A) or representative of 3 (B) replicates. Statistical analysis was performed by 1 way Anova followed by Tukey's multiple comparison tests. The following symbols indicate significant differences ($p<0.05$) with different groups: * (All); # (UT); ¤ (Blank); † (M); ‡ (R);¥ (C); $ (MC); Ø (MRC)

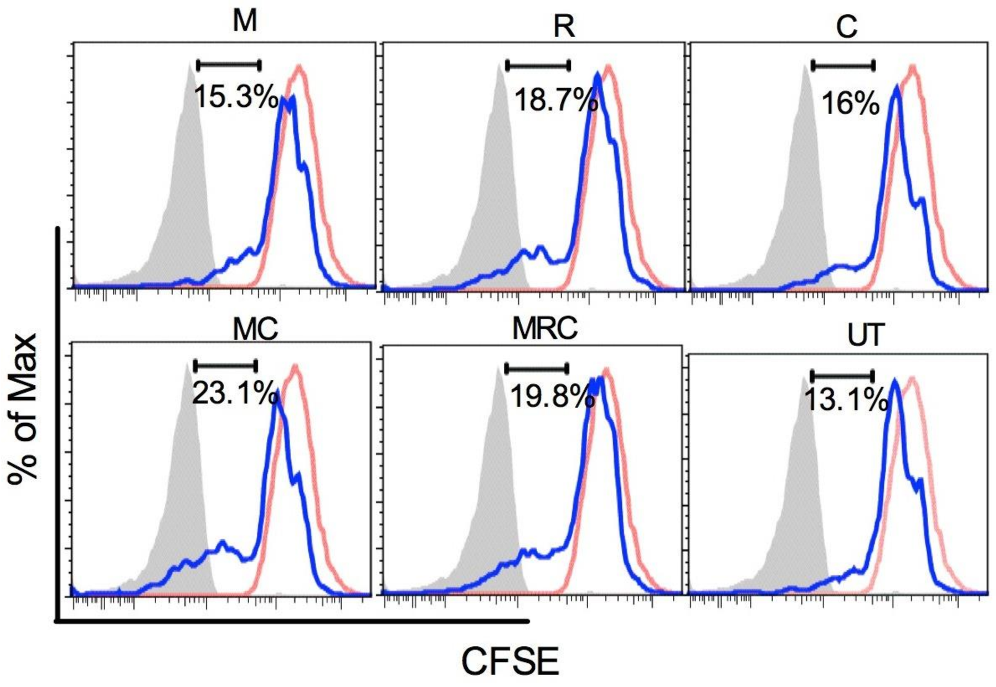

Supplementary Figure S2. CD8 T cell CFSE proliferation assay. BMDCs in medium alone (UT) or exposed to 10 ng mL^{-} of PLP-MPLA (M), 1 µg mL^{-} of PLP-R837 (R), or 100 ng mL^{-} of PLP-CpG (C), individually or in combinations of M+C or M+R+C in the presence of Ovalbumin were cocultured with CFSE labeled OTI CD8 T cells at 1:4 ratio for 72 h. Cells were stained with CD3, and CD8, and analyzed by flow Cytometry. Unlabeled (gray) and CFSE labeled and untreated (Red) T cells were used as controls. Representative histograms from 3 replicates are shown.
